# Efficacy of 8-week daclatasvir-sofosbuvir regimen in chronic hepatitis C: a systematic review and meta-analysis

**DOI:** 10.1186/s12985-024-02544-2

**Published:** 2024-11-04

**Authors:** Ahmed N. Farrag, Ahmed M. Kamel

**Affiliations:** Clinical Pharmacy Department, College of Pharmacy, Cairo University, Cairo, 11562 Egypt

**Keywords:** Hepatitis C virus, Daclatasvir, Efficacy, Shortened, 8-week

## Abstract

**Background:**

The high rates of the sustained virologic response 12 weeks after treatment (SVR12) in real world settings provoked the adoption of shortened courses of the costly direct-acting antivirals (DAAs) regimens. This study provides, to our knowledge, the first systematic review and meta-analysis for the efficacy of the shortened 8-week course of sofosbuvir (SOF) plus daclatasvir (DCV), the most accessible DAAs in the low-middle income countries (LMICs).

**Methods:**

We performed a proportion meta-analysis to determine a reliable rate of SVR12 by pooling all studies that evaluated the results of the 8-week regimen of DCV + SOF. In addition, we applied sensitivity analyses using two imputation paradigms: a conservative approach, and a pragmatic approach to avoid overestimating the efficacy of the 8-week regimen in studies that followed a response-guided treatment (RGT) approach.

**Results:**

Six studies with a total of 159 patients were included. The pooled SVR12 rate ranged from 91 to 97% in the included scenarios. The pragmatic scenario showed that the pooled SVR12 was 97% (95% confidence interval (CI) 91%; 100%) with lower variability as assessed by the prediction interval. The conservative approach revealed an SVR12 of 93% (95% CI 84%; 95%).

**Conclusion:**

The 8-week course of 60 mg DCV with SOF provided a comparable SVR12 to the standard 12-week regimen in treatment-naïve, non-HIV co-infected patients with a minimum estimated efficacy of 90%.

**Supplementary Information:**

The online version contains supplementary material available at 10.1186/s12985-024-02544-2.

## Introduction

Hepatitis C virus (HCV) infection has been a significant global health issue for decades. In 2019, there were 58 million cases and 1.5 million new infections, with over 250,000 deaths reported by the World Health Organization (WHO) [1, 2]. WHO guidelines for HCV have evolved since 2017 to accelerate the elimination of HCV by 2030 and reduce the economic burden of treatment, particularly with costly Direct-Acting Antivirals (DAAs) [2–12]. Shortened DAA regimens, supported by high sustained virologic response (SVR) rates in real-world settings, have been a key strategy [4, 13–21].

Among the DAAs currently available is the combination of sofosbuvir plus daclatasvir (SOF/DCV). SOF/DCV is approved by the European Medicines Agency (EMA) and the Food and Drug Administration (FDA), and is recommended by WHO as a pan-genotypic treatment for HCV, irrespective of the viral genotype (GT) [4, 22]. DCV is an non-structural 5 A (NS5A) inhibitor that disrupts viral replication and assembly by binding to the NS5A protein, a vital component in the HCV replication complex [13]. SOF, on the other hand, is an non-structural 5B (NS5B) polymerase inhibitor that targets the HCV Ribonucleic acid (RNA) polymerase, thereby inhibiting viral RNA replication [14]. Together, DCV and SOF act synergistically to halt the viral lifecycle, making this combination highly effective in treating HCV. In real-world practice, the SOF/ DCV regimen has demonstrated high SVR12, with success rates commonly exceeding 90% [15–21, 23].

Since 2020, SOF/DCV regimen has become the preferred DAA treatment for HCV in in low- and low-middle-income countries (LICs/LMICs) that constitute over two thirds of the global disease prevalence [24], and became one of the WHO Model List of Essential Medicines (EML) [5].

Recent studies evaluating the efficacy of the eight-week SOF/DCV regimen have indicated that this shortened course could be a viable alternative to the currently approved 12-week regimen [25–27]. However, some of these studies employed a response-guided treatment (RGT) strategy, where only patients achieving a rapid virologic response (RVR) within the first month of treatment were allocated to the shorter regimen. This approach can potentially negate the cost savings of a shorter treatment duration, as the high expense associated with HCV viral load quantification techniques poses a substantial challenge in managing HCV infection, particularly in LICs and LMICs with constrained healthcare resources [28].

In this study, we aimed to provide a current literature evaluation and, to our knowledge, the first systematic review and efficacy-adjusted meta-analysis of the shortened eight-week course of SOF/DCV for managing HCV infection, irrespective of achieving RVR.

## Methods

### Search strategy

This systematic review and meta-analysis was reported following the Preferred Reporting Items for Systematic Reviews and Meta-Analyses (PRISMA) Statement (Appendix A) [29]. The protocol was registered with the PROSPERO registry (CRD42023413487). Two independent researchers (A.K. and A.N.) searched PubMed, Scopus, and Web of Sciences for studies published before April 2023. The search strategy in each database is available in table S1 (Appendix B). All studies were exported to Mendeley (version 1.19.8, Clarivate, Philadelphia, USA). The references cited in these studies were reviewed to identify additional eligible studies. Studies included in the meta-analysis: (1) described patients with HCV treated with SOF + DCV regimen for eight weeks; (2) reported the success rate defined as SVR12 or provided sufficient data for such calculation; (3) were published in English.

The following studies were excluded: (1) case reports, letters, reviews, in vitro investigations, animal studies, and technical reports; (2) did not disclose the data necessary for the meta-analysis; (3) had duplicated samples; (4) were not written in English; and (5) were methodologically faulty (Table S2 and Figure S1). A.K. and A.N. independently assessed the study titles and abstracts to determine eligibility. The listed studies’ full texts were retrieved and evaluated.

### Quality assessment

The quality of the included studies was assessed by A.K. and A.N. using the Joanna Briggs Institute (JBI) Critical Appraisal Checklist for case series. The JBI checklist for case series includes ten items that evaluate the inclusion criteria, method of condition measurement, the validity of the diagnostic methods, consecutive enrollment of participants, adequacy of participants’ inclusion, the presentation of the demographic characteristics, clinical information, outcomes, presenting clinic demographic information and the appropriateness of the statistical methods [30].

### Data extraction

Data extraction and cross-checking were conducted by A.K. and A.N. independently. The following data were extracted: first author, publication year, age, study design, study population, prior treatment experience, cirrhosis state, baseline log_10_ HCV, the GT of the infected patients, SOF, and DCV doses, HCV assay technique applied with its Lower Limit Of Detection (LLOD) and Lower Limit Of Quantification (LLOQ), retreatment outcome in case of SVR12 failures. The primary outcome of the current study was the SVR12, defined as HCV RNA below LLOD 12 weeks following the eight weeks course of SOF + DCV.

### Data synthesis

A meta-analysis of proportions using the random-effects model was used to pool the SVR12 estimates from different studies. Without appropriate data transformation, the accompanying meta-analyses experience threats to statistical conclusion validity [31], such as the confidence limits falling outside of the established zero-to-one range and variance instability [32]. While the logit transformation solves the problem of confidence interval estimates falling outside the zero to one range, it does not necessarily resolve the issues regarding variance from extreme proportional datasets. As the double arcsine transformation (Freeman-Tukey transformation) addresses both problems listed above, it is the preferred transformation method and was implemented in the current analysis. Once the meta-analysis had been performed on the transformed proportions, a back-transformation was performed. There is still no consensus about the back-transformation method that should be used with the Freeman-Tukey double arcsine method, although the harmonic mean was suggested for back-transformation [33].

### Heterogeneity between studies

The I^2^ statistic was used to explore the percentage of heterogeneity attributed to variation in true-effect sizes secondary to inter-population variation. Estimates from subgroups within the same study were pooled using a fixed-effects model and used in the meta-analysis. The 95% confidence interval (CI) and Z-statistic were calculated and used for hypothesis testing.

### Prediction interval

The prediction interval was used to assess the treatment effect that may be predicted in future analyses, considering the different settings across different studies. It captures the variability in the true treatment effect across different settings. With substantial heterogeneity, prediction intervals will be broader than confidence intervals and might be considered a more conservative technique to integrate uncertainty in the analysis [34].

### Subgroup analysis

Subgroup analysis was performed based on HCV-GT, HCV-HIV coinfection status, DCV dose, prior HCV treatment, presence of cirrhosis, and risk of bias to explore possible sources of heterogeneity between studies.

### Publication bias

Funnel plots were used to assess publication bias, while Egger’s test was used to test the asymmetry of funnel plots [35]. The trim-and-fill method was used to detect and adjust for publication bias [36].

### Sensitivity analysis

The leave-one-out sensitivity analysis was used to assess the effect of individual studies on the observed effect size and heterogeneity. Leave-one-out sensitivity analysis was also used to assess the effect size and between-study heterogeneity after the exclusion of individual studies.

To ensure the robustness of our meta-analytic findings on the efficacy of the SOF/DCV combination for HCV treatment, we conducted data-driven sensitivity analyses. These analyses aimed to assess how varying assumptions and potential study biases impact our overall results. We employed both conservative and pragmatic scenarios to estimate the SVR12 in different patient subgroups.

In studies that adopted the RGT approach and used the RVR to assess whether patients would receive an 8-week course or a standard 12-week regimen, we proposed two scenarios to avoid overestimating the SVR12 rate in patients who achieved RVR and were assigned to the shortened 8-week regimen [37, 38]. In one scenario, we treated these patients who failed to achieve RVR and completed the 12-week regimen as SVR12 failures under the 8-week regimen (intention-to-treat scenario or conservative approach). In the second scenario, we used the lower confidence interval limit for the SVR12 (80%) from Flower’s study [25] (where RVR achievers were assigned to the 4-week arm while RVR non-achievers were assigned to the 8-week arm). The latter aims to provide a more prudent estimate for the SVR12 rate for those who failed to achieve a RVR and were accordingly assigned to the 12-week regimen instead of the 8-week one (pragmatic or data-driven approach).

### Statistical analysis

Statistical analysis was performed using R v 3.6.3 (R Core Team, Vienna, Austria) [39]. The random-effects model (using the maximum likelihood estimator for tau) was used to pool the effect sizes from the included studies. The underlying hypothesis for adopting the random-effects model is that heterogeneity or observed variance of effect is a sum of sampling error and variation in true-effect sizes stemming from inter-population variability. The generic inverse variance method was used for weighing using the per-protocol population of each trial. The Hartung-Knapp adjustment was used to prevent counterintuitive effects and to yield more conservative inferences, as the heterogeneity variance estimate is commonly associated with substantial uncertainty, especially in contexts where only a few studies are available. Forest plots were used to visualize the results. P values < 0.05 were considered statistically significant.

## Results

### Study selection

**Fig. 1:**
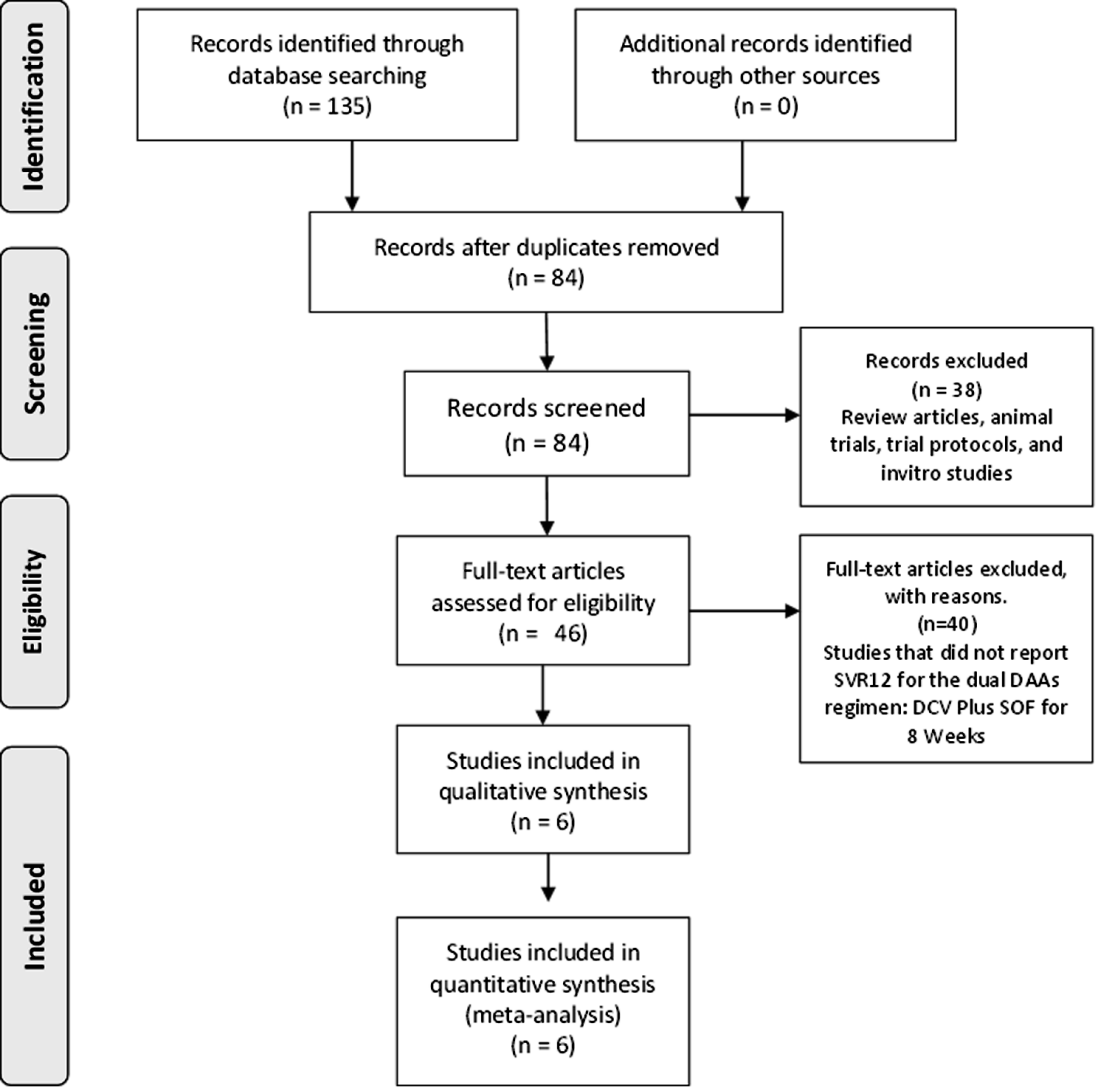
Flow chart of the search strategy for the studies included in this review and meta-analysis

After duplicates removal, eighty-four results were obtained. A.N. and A. K. screened the abstracts for clinical studies that investigated the 8-week regimen of SOF/DCV (Fig. 1). Forty-six studies were selected for further full-text assessment. After a critical appraisal of the articles, six studies were included in the qualitative and quantitative data synthesis.

A summary of the publications excluded and a full list of these publications are provided in Tables S2 and S3, respectively. Four out of the six studies included in the qualitative and quantitative analysis showed a case-series design [33]. Two studies - [26] and [27] - followed a RGT approach where patients were assigned to the shortened 8-week course if they achieved a RVR on days 2, 14, and 28 [37, 38]. Only one study [25] reported the retreatment results in patients who did not achieve SVR12. None of the included studies investigated the shortened course of SOF/DCV in HCV GT-5. Study designs and patient characteristics for the included studies are summarized in Table 1.

**Table 1:** Characteristics of the included studies

Study			Population	Design	*N* (PP)	SVR12	SVR12 per Cirrhotic status		Age ^a^ (years)	HCV Genotype						SOF (mg)	BL HCV (log_10_)	HCV RNA quantification	LLOQ/ LLOD (IU/ml)	Retreatment Course (SVR12)
										GT-1	GT-2	GT-3	GT-4	GT-6	GT -NA					
Ref	First Author	Date																		
							NC	C												
[25]	Flower B. et al.	**2023**	HCV infected patients with mild liver disease.	single arm	17	**100%** (17/17)	17/17	-	49.5 (25–67)	6/6	-	-	-	11/11	-	400	6.70	COBAS AmpliPrep/COBAS TaqManHCV Quantitative Test, v. 2.0	**LLOQ**: 15	SOF + DCV 12 weeks, (100%, 13/13) ^b^
[56]	Goel A. et al.	**2021**	Acute HCV infected patients with advanced renal failure	single arm	26	**96.2%** (25/26)	25/26	-	36 (18–74)	6/6	-	10/10	2/2	-	8/9	200	5.51	COBAS^®^ AmpliPrep/COBAS^®^ TaqMan^®^ quantitative test, v2.0	**LLOD**: 15	Not reported
[26]	El-Shabrawi M. et al.	**2018**	NC adolescent HCV infected patients	single arm	10	**100%** (10/10)	10/10	-	16 (13–17)	-	-	-	10/10	-	-	400	5.66	COBAS^®^ AmpliPrep/COBAS^®^ TaqMan^®^ HCV quantitative test, v2.0	**LLOD**: 10	Not Reported
[27]	Yakoot M. et al.	**2017**	NC chronic HCV infected patients	RCT	59 ^c^ (47 ^d^)	**100%**^e^ (47/47) **96.7%** ^**f**^ (57/59) **80%** ^**g**^ (47/59)	47/47	-	45.4 ± 7.86 ^h^	-	-	-	47/47	-	-	400	6.12	COBAS Amplicor 2.0, Roche Molecular Diagnostics	**LLOD**: 10	Not reported
[40]	Hezode C. et al.	**2017**	Treatment-naïve NC HCV infected patients	single arm	26	**92.3%** (24/26)	24/26	-	50 (36–56)	-	-	24/26	-	-	-	400	5.83	NA	NA	Not reported
[41]	Wyles D. et al. (Pooled)	**2015**	HCV/HIV patients	RCT	48	**79.2%** (38/48)	27/33	11/15	51 (28–75)	31/41	5/6	2/3	-	-	-	400	6.44	COBAS TaqMan HCV test, v. 2.0	**LLOQ**: 25 **LLOD**: 20	Not reported
	60 mg	**2015**			10	**90%** (9/10)	9/10	0	51 (40–75)	8/8	0/1	1/1	-	-	-	400	NA	COBAS TaqMan HCV test, v. 2.0	**LLOQ**: 25 **LLOD**: 20	Not reported
	30 mg	**2015**			29	**72.4%** (21/29)	10/14	11/15	NA	-	-	-	-	-	-	400	NA	COBAS TaqMan HCV test, v. 2.0	**LLOQ**: 25 **LLOD**: 20	Not reported
	90 mg	**2015**			9	**88.9%** (8/9)	8/9	0	NA	-	-	-	-	-	-	400	NA	COBAS TaqMan HCV test, v. 2.0	**LLOQ**: 25 **LLOD**: 20	Not reported

Ref, Reference; PP, Per-protocol; SVR12, Sustained virological response 12 weeks post treatment completion; NC, Non-cirrhotic; C, Cirrhotic; GT, Genotype; NA, Not available/applicable; LLOQ, Lower limit of quantification; LLOD, Lower limit of detection; BL, Baseline; RCT, Randomised controlled trial; HIV, Human immunodeficiency virus

a- Median (IQR) unless otherwise specified

b- all SVR12 failures were on the 4-week arm

c- Total number of patients in the response-guided arm

d- Actual number of patients who received the 8-week regimen

e- SVR12 based on the actual number of patients who received the 8-week regimen (per-protocol scenario)

f- SVR12 based on the predicted SVR12 in patients who received the 12-week regimen at the lower 95% confidence interval boundary for the SVR12 achieved in the 8-week regimen by Flower B. et al., 2023 (pragmatic scenario)

g- SVR12 based on the assumption that all patients who did not receive SOF + DCV for 8-week are considered as SVR12 failures (conservative scenario)

h- Mean ± SD

### Risk of bias assessment

One study was unclear regarding the statistical analysis used [40]. Two studies showed bias in terms of including patients, where only those who developed RVR were assigned to the shortened regimen of DCV + SOF (8-week regimen) [26, 27]. However, these studies adhered to our inclusion/exclusion criteria, reporting the SVR12 rate following an 8-week treatment with the dual SOF + DCV, along with patient demographics and characteristics. Both reviewers agreed to include all six studies that passed the initial inclusion/exclusion criteria screening (Table 2).

**Table 2:** Risk of bias assessment as per the JBI 2017 critical appraisal checklist for case series [30]

First author, Year [Reference]	Flower B. et al., 2023 [25]	Goel A. et al., 2021 [56]	El-Shabrawi M. et al., 2018 [26]	Yakoot M. et al., 2017 [27]	Hezode L. et al., 2017 [40]	Wyles D. et al., 2015 [41]
1. Were there clear criteria for inclusion in the case series?	Yes	Yes	Yes	Yes	Yes	Yes
2. Was the condition measured in a standard, reliable way for all participants included in the case series?	Yes	Yes	Yes	Yes	Yes	Yes
3. Were valid methods used for identification of the condition for all participants included in the case series?	Yes	Yes	Yes	Yes	Yes	Yes
4. Did the case series have consecutive inclusion of participants?	Yes	Yes	**No**	**No**	Yes	Yes
5. Did the case series have complete inclusion of participants?	Yes	Yes	Yes	Yes	Yes	Yes
6. Was there clear reporting of the demographics of the participants in the study?	Yes	Yes	Yes	Yes	Yes	Yes
7. Was there clear reporting of clinical information of the participants?	Yes	Yes	Yes	Yes	Yes	Yes
8. Were the outcomes or follow up results of cases clearly reported?	Yes	Yes	Yes	Yes	Yes	Yes
9. Was there clear reporting of the presenting site(s)/clinic(s) demographic information?	Yes	Yes	Yes	Yes	Yes	Yes
10. Was statistical analysis appropriate?	Yes	Yes	Yes	Yes	**Unclear**	Yes
Overall appraisal	Include	Include	Include	Include	Include	Include

### Study outcomes

**Fig. 2:**
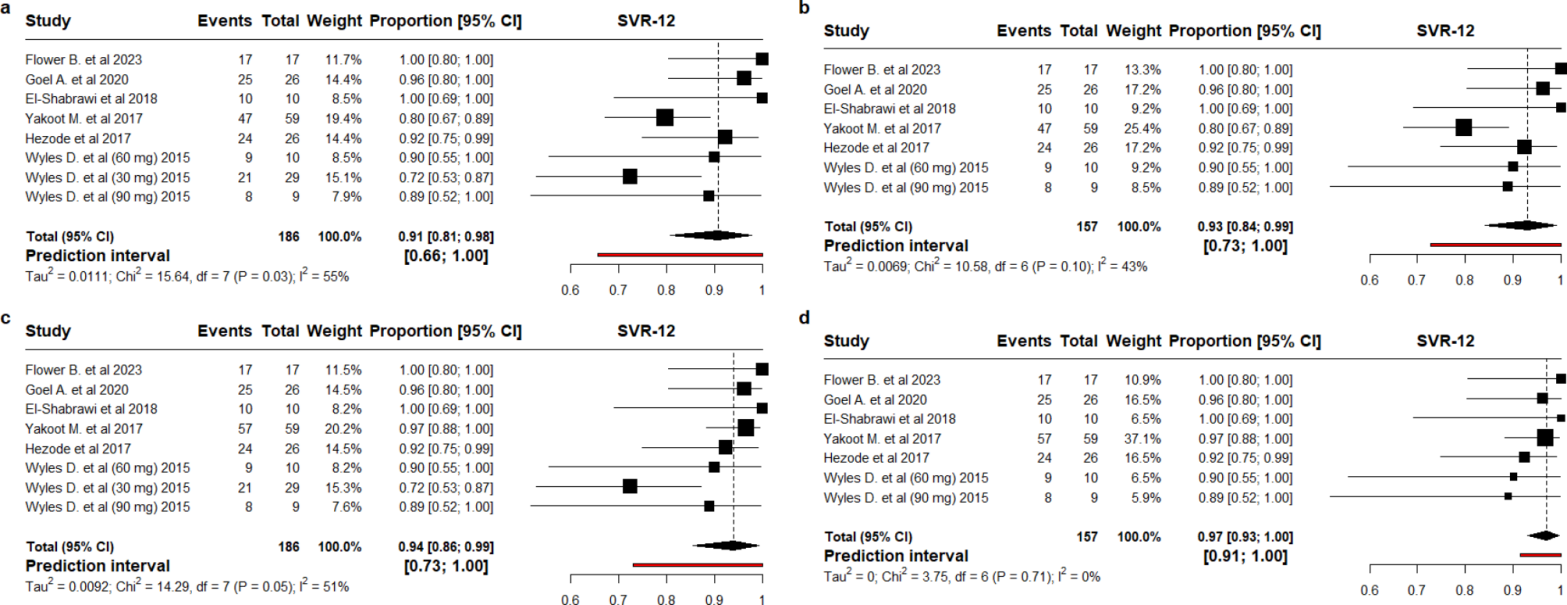
Meta-analysis of SVR12 rates based on four scenarios (**a**) Including all studies and including patients who did not achieve RVR as failures, (**b**) Scenario (**a**) plus excluding HIV patients who received DCV 30 mg, (**c**) using the lower margin of success for patients who did not achieve RVR, (**d**) scenario (**c**) plus excluding patients who received DCV 30 mg. CI: Confidence interval; SVR12: HCV RNA 12 weeks after the end of treatment (SVR12); DCV: Daclatasvir

The pooled SVR12 rate varied between 91% and 97% across the included scenarios (Fig. 2). In the conservative scenario, where the SVR12 rate in the study conducted by Yakoot M [27] was estimated to be 78% (46/59), the pooled SVR12 rate across all DCV dose regimens (30 mg, 60 mg, 90 mg) was 91% (95% CI 81%; 98%) (Fig. 2a), and 93% (95% CI 84%; 99%) for only the 60 mg DCV dose (Fig. 2b) with heterogeneity decreasing from 55 to 43%.

In the pragmatic scenario, where we estimated the SVR12 rate in the study conducted by Yakoot M [27] to be 97% (57/59), the pooled SVR12 rate was 94% (95% CI 86%; 99%) for all doses of DCV (Fig. 2c), and 97% (95% CI 93%; 100%) for the 60 mg DCV (Fig. 2d). The SVR12 rate for the other study which used RGT [26] remained unchanged as no patients failed to reach an RVR in that study. Applying such an approach, where it was assumed that not all patients failing to achieve RVR failed treatment (Fig. 2c), heterogeneity in the SVR12 estimates decreased from 55 to 51%.

Interestingly, combining both approaches in the fourth scenario (Fig. 2d), where we excluded patients receiving 30 mg DCV and assumed an 80% SVR12 for those not on the 8-week course (pragmatic scenario), no heterogeneity was observed (I^2^ = 0) and a more precise estimate was obtained (Pooled SVR12 = 97%, 95% CI 91%; 100%) with less variability (prediction interval 91-100%).

**Fig. 3:**
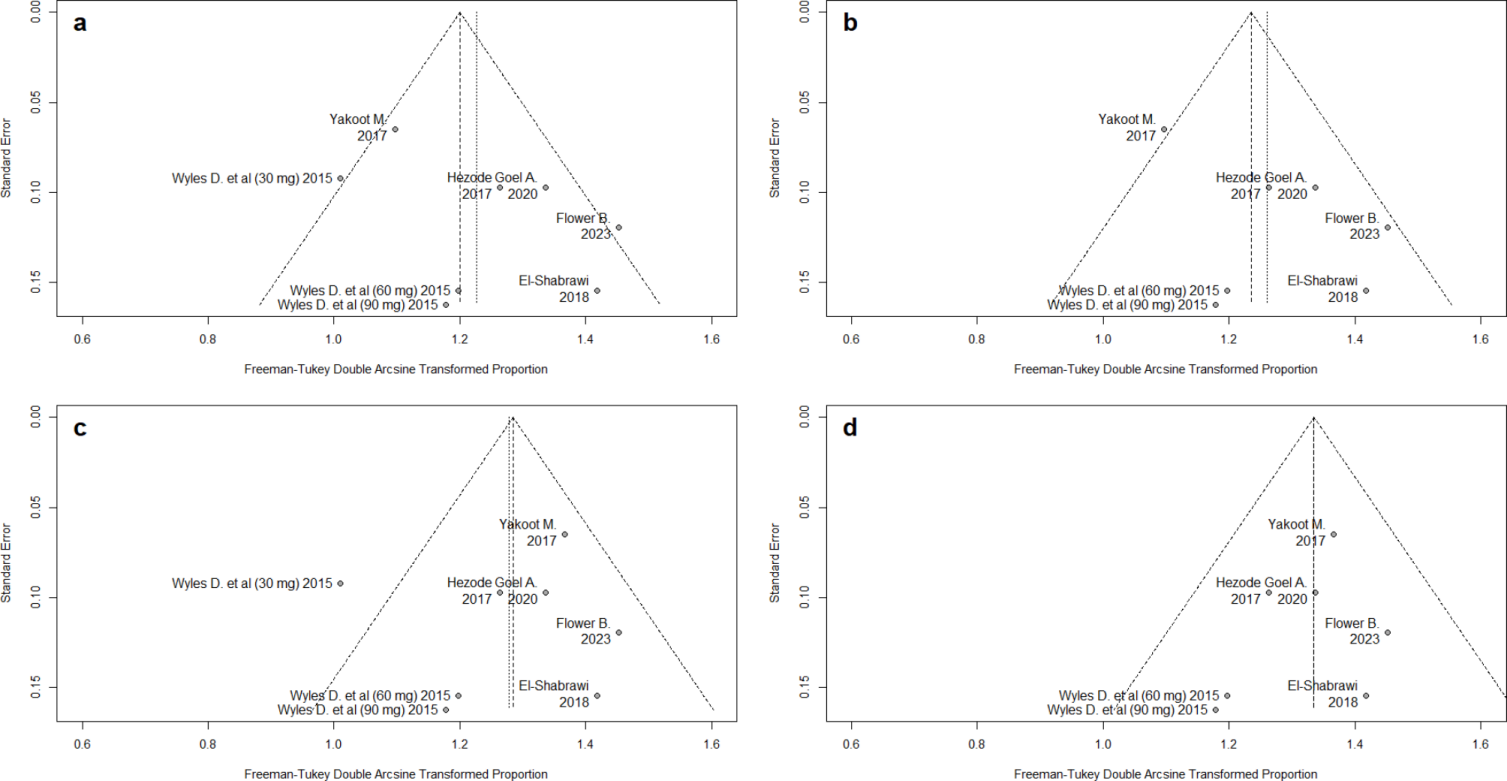
Publication bias for the included four scenarios (**a**) scenario a (**b**) scenario b, (**c**) scenario c, and (**d**) scenario d

The publication bias analysis, using the Freeman-Tukey double arcsine transformation, indicated that the imputed SVR12 rate under the pragmatic assumption was more unbiased and consistent with other study reports in this review (Fig. 3). The hypothesis that the 30 mg DCV subgroup in Wyles D conveyed a biased SVR12 estimate was also supported by the publication bias analysis, where a symmetrical funnel plot is produced once this subgroup was excluded (Fig. 3b and Fig. 3d). The exclusion of the 30 mg arm in Wyles D is clinically justified, since this dosing has become obsolete for HCV patients without HIV co-infection and not on concurrent ritonavir-boosted protease inhibitors.

**Fig. 4:**
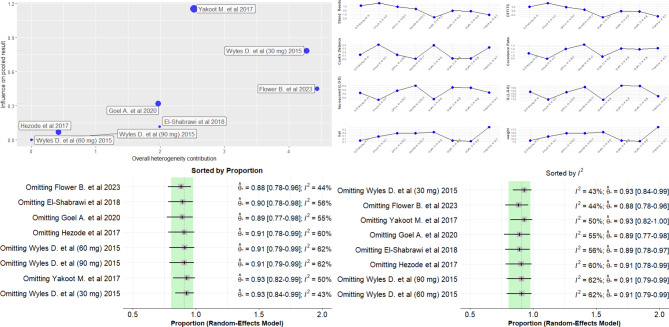
Influence analysis results using scenario (a)

Egger’s test did not indicate statistically significant publication bias in any scenario, though the low number of studies prevents a definitive conclusion (Fig. 3). No publication bias was observed in the conservative scenario with small studies showing high effect sizes and standard errors (Fig. 3a). The studies by Yakoot M [27] and a subgroup from Wyles D study (30 mg dose) [41] had small effect sizes and standard errors in scenarios (a) - Fig. 3a, and (b) - Fig. 3c. In scenario (c), the 30 mg DCV subgroup was the only identified source of heterogeneity, while a symmetrical funnel plot appeared in scenario (d). Leave one out sensitivity analysis showed that the study conducted by Yakoot M [27] had the biggest influence on heterogeneity (Fig. 4). Omitting either study conducted by Yakoot M [27] or Wyles D [41] (only patients who received 30 mg) resulted in a pooled SVR12 of 93%, a more precise estimate, and a reduction in between-study heterogeneity to 43% and 50%, respectively.

Subgroup analysis showed that HIV status was significantly associated with the pooled estimate for SVR12 (P interaction < 0.01) as shown in Figure S1. The pooled estimate for HIV-positive patients was lower than that observed in HIV-negative patients (97% vs. 80%). Similarly, DCV was significantly associated with the pooled estimate for SVR12 (*P* < 0.01). The pooled SVR12 in patients who received 60 mg of DCV daily was higher than that in patients who received 30 mg (97% vs. 72%), with no heterogeneity observed between studies that used 60 mg of DCV. No interaction was observed between GT and the pooled SVR12 estimate (P _interaction_ = 0.21). Prior treatment status was not significantly associated with the pooled SVR12 estimate (P interaction = 0.17). Cirrhosis was associated with a lower pooled SVR12 estimate (73% vs. 95% in cirrhotic and non-cirrhotic patients, respectively, P interaction = 0.03). It should be noted that only one study included a subgroup of patients with cirrhosis.

The eight-week regimen of SOF/DCV (60 mg), which precludes patients who received 30 mg [42] and imputes the SVR12 at the lowest reported margin [27], showed the most plausible and unbiased picture that reflects the efficacy of the 8-week course of SOF/DCV (60 mg) in HCV treatment-naïve patients without HIV coinfection. This scenario showed a mean SVR12 of 97% (95% CI: 93–100%) and offered a remarkable reduction in heterogeneity score.

## Discussion

The revolutionary introduction of the novel DAAs to the battle of HCV combat made the elimination of HCV a much more attainable pursuit than before. Since its first release in 2011, DAAs resulted in considerably higher SVR12 cure rates at shorter duration and much more desirable safety profiles than classical interferon-based regimens [43]. The accumulated data on the high efficacy of the shortened regimen of Glecaprevir + Pibrentasvir prompted the FDA to expand its licensed regimen from 12 weeks to only eight weeks in candidate HCV patients [44]. Recent reports on the SOF-based regimens provided more evidence on the efficacy of the shortened eight-week course of many of its dual combinations, including SOF/LDV, SOF/Velpatasvir, and SOF/DCV [45–51].

The efficacy of the shortened 8-week course of DCV + SOF was initially supported using HCV viral kinetic modeling [52]. According to the model, the extrapolated time-to-cure (TTC)—the duration needed to reduce virions in the extracellular fluid to less than one, thereby eliminating the risk of latent viral replication and disease relapse—was predicted to be less than 8 weeks [53].

The clinical efficacy of the shortened 8-week course of DCV + SOF was first questioned by Wyles D [42] in the ALLY-2 trial. Although it highlighted a low SVR12 of the pooled 8-week regimen (76%), it implicitly outlined a promising efficacy for the 8-week course of the standard 60 mg dose of DCV. This efficacy is reflected by the fact that the fraction of patients who received the regular 60 mg DCV dose for eight weeks and achieved SVR12 is 9/10 (90%). The remaining SVR12 failures in this arm were observed with the reduced DCV dose (30 mg), and only one failure was linked to the 90 mg dose. The 30 mg dose of DCV in the 8-week arm was based on the pharmacokinetic drug-drug interaction between DCV and the ritonavir-augmented antiretroviral medications [54].

Later, Hezode C [40] evaluated the 60 mg dose of DCV in an 8-week regimen for chronic HCV GT-3 infection, achieving an SVR12 of 92% (24/26), comparable to the 89% (797/895) achieved with the 12-week regimen for GT-3 [55]. Further supporting this regimen, El- Shabrawi, M [26] reported a 100% SVR12 (9/9) in adolescents infected with HCV GT-4, compared to 97% (178/183) with the standard regimen [55].

In another study, Flower B [25] documented a 100% SVR12 (17/17) for GTs 1 and 6 using the 8-week SOF/DCV regimen [25, 51, 55]. Additionally, Goel A [56] observed a 100% SVR12 (15/15) following the shortened 8-week course of DCV (60 mg) plus half the regular dose of SOF (200 mg) in patients with acute HCV infection and renal impairment, comparable to the 97.2% (35/36) achieved with the standard regimen in hemodialysis patients [57].

Although previous studies suggest that RVR does not provide a clinically reliable predictor of SVR12 [58–60], two out of the six studies in this review [26, 27] followed a RGT approach, where only patients who showed a RVR on days 2, 14, or 28 after treatment initiation were assigned to the shortened regimen. RGT, using on-treatment HCV-RNA, was historically used to predict response to IFN-based therapy and optimize treatment duration, especially for telaprevir- and boceprevir-based therapies. However, the high effectiveness of IFN-free regimens, with SVR rates exceeding 90–95%, has diminished the need for RGT [61]. Furthermore, this approach can offset the cost savings derived from a shortened treatment regimen as the high expense of HCV-RNA quantification techniques remains a significant hurdle in effectively managing hepatitis C, especially in LICs and LMICs with limited healthcare resources [62]. To ensure these regions can implement and sustain treatment programs, it is crucial to adopt strategies that minimize the frequency of costly viral load monitoring tests [63]. By optimizing treatment protocols to include only the essential tests—preferably at baseline for definitive diagnosis, weeks 12 and 24 after starting treatment to confirm a cure—hepatitis C management can become more accessible and affordable.

In this context, and to derive more unbiased estimates resulting from the two RGT studies in our meta-analysis, we conducted sensitivity analyses, influence analysis, and publication bias analysis. These analyses employed both conservative and pragmatic scenarios to avoid overestimating the SVR12 rate in patients who achieved RVR and were assigned to the shortened 8-week regimen.

Our findings showed that the 8-week regimen using the standard dose of DCV (60 mg) + SOF resulted in a mean SVR12 of 97% (95% CI: 93–100%) and a prediction interval (95% CI: 91–100%), with no heterogeneity between the effect size reported in the different studies. We believe these results from the pragmatic scenario offer the most credible and unbiased estimate of the efficacy of the 8-week course of 60 mg SOF/ DCV in clinical practice.

Additionally, our subgroup analyses (Figure S1 - Appendix B) revealed statistically significant lower SVR12 rates in cirrhotic patients (*P* = 0.03), patients with HIV coinfection (*P* < 0.01), who received a 30 mg DCV dose (*P* < 0.01).

The lower SVR12 rates observed in HIV-coinfected patients may be attributed to the reduced 30 mg dose of DCV used in this population, rather than the coinfection itself. However, this association is not definitively established, while the reduced SVR12 observed in cirrhosis is consistent with the reported categorization of cirrhotic patients as a difficult-to-treat subgroup. Thus, it is recommended that these patients should receive either SOF/DCV for 24 weeks or SOF/ DCV/Ribavirin for 12 weeks as per standardized protocols [63].

Concerning treatment failures associated with the shortened course of SOF/DCV, all patients who did not achieve SVR12 with a four-week regimen in Flower B [25] were successfully cured when retreated for 12 weeks with the same DAA combination (100%). This outcome aligns with real-world data on the efficacy of retreatment in SOF/DCV relapsers or non-responders, who achieved an SVR12 success rate of 92.7% with other DAA regimens [64].

From a pharmacoeconomic perspective, cost-effectiveness analyses comparing shortened DAA regimens (including SOF + DCV) to the standard 12-week regimens strongly recommended adopting 8-week regimens. This analysis mainly considered the monetary savings by cutting the cost of treatment to two-thirds, and the additional expenses incurred from subsequent retreatment protocols in case of SVR12 failures [65].

This meta-analysis does have some limitations. First, although the heterogeneity in scenario-4 is reduced to near null, the number of studies included in the meta-analysis is relatively small, which limits the ability to derive a broader perspective on the 8-week regimen’s efficacy. Second, even though our meta-analysis utilized arcsine transformation to account for the single-arm design of the studies in this review, there is a strong need for a randomized controlled trial (RCT) in which HCV patients are randomly assigned to either the 8-week regimen or the standard 12-week course, regardless of their baseline viral load or early virological response. Third, the trim-and-fill method used to identify and correct for publication bias may underestimate the true effect when there is substantial between-study heterogeneity (variability in the true effect size), even if publication bias is not present [66]. Fourth, given the lack of comprehensive SVR12 data in cirrhotic patients, particularly those with decompensated cirrhosis, our results cannot be generalized to this specific subgroup. Similarly, our findings on the efficacy of the 8-week regimen of DCV should not be extrapolated to HIV-coinfections. While all patients who received the 30 mg dose of DCV were HIV-coinfected, it remains unclear whether the low SVR12 rates observed in those patients were due to the reduced DCV dose, the HIV coinfection, or both. This relationship needs to be clearly established before drawing conclusions. Finally, none of the studies included in this review investigated GT-5, which limits the relevance and applicability of our findings to this specific genotype. Nevertheless, HCV GT-5 has shown high SVR12 rates with various shortened DAA regimens and accounts for fewer than 1% of HCV cases globally [66, 67].

## Conclusion

In conclusion, our results show that the 8-week course of 60 mg DCV + SOF provided a comparable alternative to the standard 12-week regimen in treatment-naïve, non-HIV co-infected HCV patients with a minimum estimated efficacy of 90%.

## Electronic supplementary material

Below is the link to the electronic supplementary material.

Supplementary Material 1

Supplementary Material 2

## Data Availability

Data to support the research findings are available from the corresponding author upon request.

## Abbreviations

AASLD: American Association for the Study of Liver Diseases
CI: Confidence Interval
DAA: Direct-Acting Antiviral
DCV: Daclatasvir
EASL: European Association for the Study of Liver
EMA: European Medicines Agency
EML: WHO Model List of Essential Medicines
FDA: U.S. Food and Drug Administration
GT: Genotype
HIV: Human Immunodeficiency Virus
LDV: Ledipasvir
LMICs: Low-Middle Income Countries
LLOD: Lower Limit Of Detection
LLOQ: Lower Limit Of Quantification
MPP: Medicines Patent Pool
NICE: British National Institute for Health and Care Excellence
NS5A: Non-structural 5 A
NS5B: Non-structural 5B
PRISMA: Preferred Reporting Items for Systematic Reviews and Meta-Analyses
RCT: Randomized Controlled Trial
RGT: Response-Guided Treatment
SOF: Sofosbuvir
SVR: Sustained Virologic Response
TTC: Time-To-Cure
