# Supplementary material for: Efficacy of 8-week daclatasvir-sofosbuvir regimen in chronic hepatitis C: a systematic review and meta-analysis
Source: Virol J. 2024 Nov 4;21:275. doi: 10.1186/s12985-024-02544-2 (PMC11533316; doi:10.1186/s12985-024-02544-2)
Supplement: Supplementary file 2 — Supplementary Material 2 [file 12985_2024_2544_MOESM2_ESM.docx]

| **Appendix B** | |
| --- | --- |
| **Table S1:** Search strategy | **2** |
| **Table S2.** Summary of the publications excluded | **3** |
| **Figure S1.** Subgroup analysis results | **4** |
| **Figure S2.** Full list of the publications excluded | **5** |

**Table S1:** Search strategy

| **PubMed** | **Scopus** | **Web Of Sciences** |
| --- | --- | --- |
| (Daclatasvir[Title/Abstract]) **AND**  (("8 weeks"[Title/Abstract]) **OR**  ("8-weeks"[Title/Abstract]) **OR**  ("8 week"[Title/Abstract]) **OR**  ("8-week"[Title/Abstract]) **OR**  ("eight week"[Title/Abstract]) **OR**  ("eight-week"[Title/Abstract]) **OR**  ("eight weeks"[Title/Abstract]) **OR**  ("eight-weeks"[Title/Abstract]) **OR**  ("two months"[Title/Abstract]) **OR**  ("two-months"[Title/Abstract]) **OR**  ("two-month"[Title/Abstract]) **OR**  (“fifty-six days”[Title/Abstract]) **OR**  (“56 days”[Title/Abstract]) **OR**  (“response guided”[Title/Abstract]) **OR**  (“response-guided”[Title/Abstract]) **OR**  (“shortened”[Title/Abstract])) | (TITLE-ABS (Daclatasvir)) **AND**  ((TITLE-ABS ("8 weeks") **OR**  (TITLE-ABS ("8-weeks”) **OR**  (TITLE-ABS ("8 week") **OR**  (TITLE-ABS ("8-week”) **OR**  (TITLE-ABS ("eight week") **OR**  (TITLE-ABS( "eight-week") **OR**  (TITLE-ABS ("eight weeks") **OR**  (TITLE-ABS ("eight-weeks") **OR**  (TITLE-ABS ("two months”) **OR**  (TITLE-ABS ("two-months") **OR**  (TITLE-ABS ("two-month") **OR**  (TITLE-ABS (“fifty-six days”) **OR**  (TITLE-ABS (“56 days”) **OR**  (TITLE-ABS (“response guided”) **OR**  (TITLE-ABS (“response-guided”) **OR**  (TITLE-ABS (“shortened” )) | (AB=(Daclatasvir)) **AND**  ((AB= ("8 weeks") **OR**  (AB=("8-weeks”) **OR**  (AB=("8 week") **OR**  (AB=("8-week”) **OR**  (AB=("eight week") **OR**  (AB=( "eight-week") **OR**  (AB=("eight weeks") **OR**  (AB=("eight-weeks") **OR**  (AB=("two months”) **OR**  (AB=("two-months") **OR**  (AB=("two-month") **OR**  (AB=(“fifty-six days”) **OR**  (AB=(“56 days”) **OR**  (AB=(“response guided”) **OR**  (AB=(“response-guided”) **OR**  (AB=(“shortened”))) |

**Table S2**. Summary of publications excluded

| Nature of publication | Number | | |
| --- | --- | --- | --- |
| Protocols | 9 | | |
| URL link | status | Results | SOF+60 mg DCV for 8 Wks.? |
| https://trialsearch.who.int/Trial2.aspx?TrialID=ACTRN12617000263392 | completed | Yes | Yes |
| https://clinicaltrials.gov/ct2/show/results/NCT01888900 | completed | Yes | No |
| https://trialsearch.who.int/Trial2.aspx?TrialID=JPRN-UMIN000021790 | completed | Yes | No |
| https://trialsearch.who.int/Trial2.aspx?TrialID=JPRN-UMIN000019659 | completed | Yes | No |
| https://trialsearch.who.int/Trial2.aspx?TrialID=JPRN-UMIN000021544 | Ongoing | No | No |
| https://trialsearch.who.int/Trial2.aspx?TrialID=ISRCTN61522291 | Ongoing | No | No |
| https://clinicaltrials.gov/ct2/show/NCT03794258 | completed | Yes | No |
| <https://clinicaltrials.gov/show/NCT02349048> | completed | Yes | No |
| <https://clinicaltrials.gov/show/NCT025512861> | withdrawn | No | Yes |
| Meeting conferences | 1 | | |
| Review Articles | 24 | | |
| Preclinical Studies | 2 | | |
| Case reports | 2 | | |
| Studies did not include the dual DAAs:  DCV Plus SOF for 8 Weeks | 40 | | |
| Total | 78 | | |

**Figure S1.** Subgroup analysis results

**CI**, Confidence interval; **SVR12**: sustained virologic response; **DCV**: Daclatasvir; **HIV**, human immunodeficiency virus


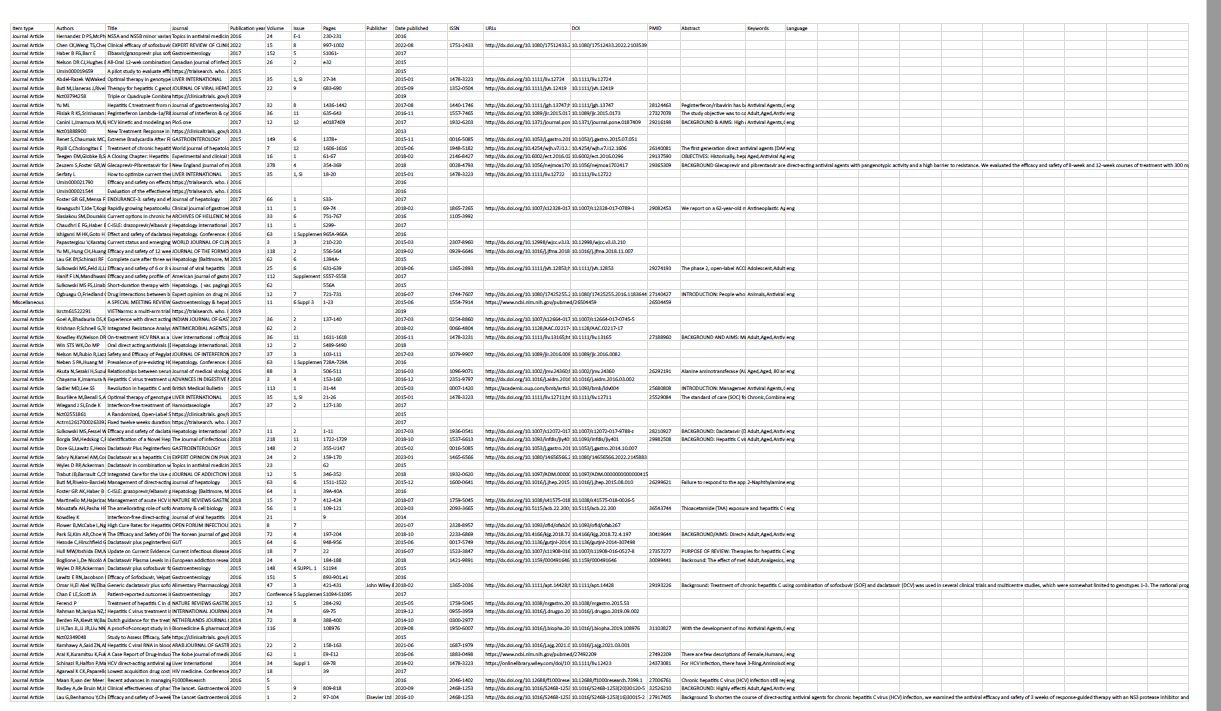


**Figure S1**. Full List of publications excluded
